# A Multimodal Network Security Framework for Healthcare Based on Deep Learning

**DOI:** 10.1155/2023/9041355

**Published:** 2023-02-20

**Authors:** Qiang Qiang Chen, Jian Ping Li, Amin ul Haq, Bless Lord Y. Agbley, Arif Hussain, Inayat Khan, Riaz Ullah Khan, Jalaluddin Khan, Ijaz Ali

**Affiliations:** ^1^School of Computer Science and Engineering, University of Electronic Science and Technology China, Chengdu 611731, China; ^2^Abdul Wali Khan University Mardan, Mardan 23200, KPK, Pakistan; ^3^Department of Computer Science, University of Buner, Buner 19290, Pakistan; ^4^Yangtze Delta Region Institute (Huzhou), University of Electronic Science and Technology of China, Huzhou 313001, China; ^5^Department of Computer Science and Engineering, Koneru Lakshmaiah Education Foundation, Guntur, Andhra Pradesh 522502, India; ^6^Iqra National University Swat Campus Odigram, Department of Computer Science, Swat 19130, Pakistan

## Abstract

As the network is closely related to people's daily life, network security has become an important factor affecting the physical and mental health of human beings. Network flow classification is the foundation of network security. It is the basis for providing various network services such as network security maintenance, network monitoring, and network quality of service (QoS). Therefore, this field has always been a hot spot of academic and industrial research. Existing studies have shown that through appropriate data preprocessing techniques, machine learning methods can be used to classify network flows, most of which, however, are based on manually and expert-originated feature sets; it is a time-consuming and laborious work. Moreover, only features extracted by a single model can be used in classification tasks, which can easily make the model inefficient and prone to overfitting. In order to solve the abovementioned problems, this study proposes a multimodal automatic analysis framework based on spatial and sequential features. The framework is completely based on the deep learning method and realizes automatic extraction of two types of features, which is very suitable for processing large-flow information; this improves the efficiency of network flow classification. There are two types of frameworks based on pretraining and joint-training, respectively, with analyzing the advantages and disadvantages of them in practice. In terms of evaluation, compared with the previous methods, the experimental results show that the framework has good performance in both accuracy and stability.

## 1. Introduction

The rapid development of the Internet makes the Internet technology penetrate into all aspects of people's lives. The quality of the network environment is closely related to the physical and mental health of human beings. At present, various types of bad website traffic or apps will push bad information or use network viruses to infringe on privacy, so it is very important to classify various network applications in a timely and effective way. Network flow classification refers to the use of a certain algorithm to construct a classification model, which can be used to classify network flow of various applications. It is a fundamental work for providing various network services such as network security, network monitoring, and quality of service (QoS). Therefore, this field has always been a hot spot in academic and industrial research [1]. With the continuous development of the Internet of Things, many devices are connected to the network, and network flow classification has become an important part of this scenario [2]. New network applications based on different devices are emerging with the mutual information interaction between applications which has prompted the network to face the status quo of large throughput and difficulty of network flow classification. It is urgent to deal with large and complex types of network flow and improve the efficiency of classification.

So far, scholars have proposed many different network flow classification techniques. These technologies are mainly divided into four categories: port-based, deep packets inspection (DPI)-based [3], machine learning (ML) [4–6], and deep learning methods (different from traditional machine learning methods, deep learning is listed separately for better discussion). On the one hand, due to the development of network technology itself, classification techniques used previously such as port detection are no longer adequate for the current network flow classification. Along with the importance of data privacy, deep packet inspection is no longer favored by researchers and engineers. With the rise and vigorous development of artificial intelligence technology, intelligent classification technology has become an important direction for researchers. Network flow classification technology based on machine learning and deep learning has emerged as the main method of current classification research. This study summarizes the different methods based on machine learning and analyzes the main methods of deep learning to propose a multimodal framework, which not only improves the classification accuracy but also enhances the stability of the model. The main contributions of this study are as follows:

- The network flow classification based on spatial features and time series features is studied by using visualization methods
- A multimodal network flow classification method is proposed, which integrates different network flow features to improve the stability and accuracy of the classification model
- a comprehensive analysis of the differences in structure and training methods of the two types of models in multimodal framework and their advantages and disadvantages and solid experiments on the ISCXVPN2016 dataset

The study is structured as follows. The second part summarizes the related work in the field of network flow classification, and the third part discusses the research methodology, including the data processing, the structure of the framework, and differences between them. The fourth part is the comparison of experimental results and analysis. The fifth part is the conclusion and future work. For the convenience of writing, the acronyms used in this article are listed in Table 1.

## 2. Related Work

As the basic task of many network services, network flow classification has always been the focus of research in academic and engineering fields. So far, network flow classification technologies are mainly divided into four categories, namely, port-based, deep packet inspection, machine learning, and deep learning method.

The earliest network flow classification technology is to use port number of UDP or TCP protocol at the transport layer. This method is easy to implement with lower algorithm complexity, so it is often used to detect certain specified port applications. However, with the diversification of applications and protocols, as well as the emergence of port hopping and port masquerading technologies, this method is no longer reliable and can only be used as an auxiliary method. Many current network applications use port numbers that are different from common ports to bypass operating system access control permissions [7], while some other network applications encapsulate different services into well-known streams such as HTTP protocol-based streams or conversation, these operations usually reduce the accuracy of port-based network flow classification [7]. In fact, Madhukar and Williamson [8] proved that nearly 70% of network flow cannot be classified correctly using the port-based identification method.

DPI refers to the identification of the unique fingerprint characteristics reflected in the payload of each packet and then the detection of specific network flow [9, 10]. If the payload of the network flow and the known application or protocol can match in certain features, then it can be considered that this network flow is the known application or protocol with a high probability. For example, some traditional load data fingerprint features include: “ \ GET”-http, “0 × 13Bit”-Bit torrent p2p, “PNG”0 × 0d0a-MSN messenger, “USERHOST”-IRC, “ARTICLE”-nntp, and “SSH”-ssh Internet traffic [11]. Compared with port-based method, its accuracy is greatly improved. Although the DPI method is very accurate for the network flow classification, it also requires scientific research staff to extract characteristic fingerprints of network flow, and to maintain and update the existing fingerprint database from time to time, which is a very resource-consuming task.

In recent years, researchers tried to use the statistical characteristics of network flow and machine learning algorithms to classify network flow. Different network flow will produce different traffic characteristics that can be used to distinguish, such as the distribution of data packet size, data packet arrival time, flow length, and flow duration. Among them, Moore and Zuev [12] proposed a method based on the naive Bayes principle which builds a Bayesian classifier for supervised learning, combining with the fast correlation-based filter (FCBF) algorithm and kernel estimation technology, the method can achieve 95% accuracy. In [13], authors used nearest neighbour and linear discriminate analysis (LDA) to classify various applications. The experimental results showed that supervised ML algorithms are also able to separate traffic into classes with encouraging accuracy. In [4], authors used Bayesian network, C4.5 decision tree, naïve Bayes, and naïve Bayes tree methods to give common features set for classification with different feature selection algorithms. In [14] authors used a variety of different algorithms to filter the wrong label data in the original data set to obtain a more accurate training data set, and used machine learning to retrain the filtered data to obtain a more accurate and stable classifier. In [15], authors extracted the unique characteristics of various application during the information commutation, and realized a lightweight classification method for network application. Although the classic machine learning has solved the problems that cannot be settled with methods based on the port or DPI, it also faces many new problems. The first problem is feature selection, as machine learning methods rely on manually and expert-originated feature sets, which requires a lot of manpower to choose a feature set by themselves. The second problem is feature extraction, as the feature set that performs well for a specific data set does not have universal applicability in practice.

With the rapid development of deep learning in the field of artificial intelligence, researchers have tried to transfer deep learning methods that shine in computer vision processing, natural language recognition to the field of network flow classification. In [16], authors used neural network (NN) and sparse autoencoder (SAE) network to classify specific network protocol traffic and achieved a high accuracy. In [17], authors explored online traffic detection methods. In this study, the basic idea is to employ a compact nonparametric kernel embedding based method to convert early flow sequences into images which can be trained in convolutional neural network (CNN) and its accuracy exceeds 99%. Classification tasks can also be accomplished using network flow sequential information [18]. In [19], authors investigated the classification and prediction performances of LSTM networks, using real server-generated traffic streams, experiment result showed that LSTM is able to classify and predict the occurrence of highly intensive traffic flows accurately. In [20], authors used CNN LSTM network and their various combinations to detect network flows, which the classification accuracy for applications reached 96%. In [21], CNN and CNN & LSTM was used to classify mobile applications where the payload of the first few packets were mainly used to achieve high accuracy. In [22], authors focused on three practical problems which are network bandwidth, network flow duration, and network flow detection and then proposed a multitask training method, that is, first used the CNN network to train the network bandwidth and duration tasks and then trained the network flow classification task. Based on this training method, it had achieved better result than the previous CNN & RNN method. In [23], authors proposed to introduce the capsule network into the field of network flow classification, and combined the advantages of CNN & LSTM network to achieve high accuracy of network classification. Giuseppe Aceto etc. in [24] provided a wide experiment analysis based on multimodal framework (CNN + LSTM) for classification of encrypted mobile traffic. This work provides guidance for the subsequent exploration of multimodel fusion. Then, in [25], authors further proposed a novel multimodel multitask deep learning approach and DISTILLER classifier, it can solve different traffic classification simultaneously. Liu et al. [26] proposed a method which applied RNN to encrypted traffic classification. Moreover, the framework added a multilayer structure which can explore sequential characteristics deeply and experiment results outperformed the state-of-the-art methods. In [27], authors tried to use explainable artificial intelligence to improve multimodel behavior, the experiment results showed that the proposed method provide global interpretation, rather than sample-based ones.  Table 2 lists an overview of the above literature citations.

## 3. Research Methodology

### 3.1. Dataset

In this article, we used two different datasets including the USTC dataset provided in [28] and the ISCXVPN2016 dataset [29] provided by the Canadian Institute for Cybersecurity. The USTC dataset has 10 categories of normal traffic such as FaceTime and Gmail generated using IXIA BPS (Professional Traffic Simulator); this study will use this dataset for feature analysis of network flow classification. The ISCXVPN2016 dataset is captured at the University of New Brunswick which contains raw pcap files of several traffic types. The dataset provides labels with different categorization, such as AIM chat, Gmail, Facebook, chat, and streaming . The ISCXVPN2016 dataset is publicly available for researchers, and this study will use this dataset to conduct experiments and compare the experimental results. For more details on the captured traffic and the traffic generation process, refer to [29].

### 3.2. Method Background

In the following sections, the background of the proposed framework is presented.

#### 3.2.1. Feature Selection and Classification

**Figure 1:**
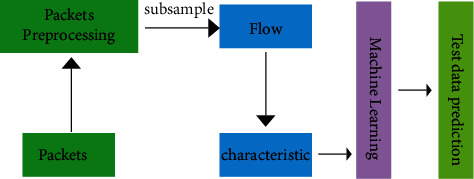
The processing of machine learning.

Network flow has an obvious hierarchical architecture: according to the general TCP/IP system structure, the network flow is packaged into data units in different layers which is unique to each layer. The frame of the data link layer is the lower-level data that can be studied which receive the data frame from the upper layer and disassemble it into data in the form of bit stream. Therefore, the frame contains different types of features in network flow that can be distinguished, so it is very important to take the frame of data link layer as the basic research object for network flow classification. In practice, the frame is easy to obtain, and all the protocol packets can be directly captured which are passing through the network card. For example, using wireshark and tcpdump can capture any data packet of interest. In the field of traffic classification, the usage of machine learning methods based on traffic characteristics has greatly improved the accuracy of classification compared with the previous methods [12, 30]. Research on this type of method shows that the key to improve the accuracy of network flow classification lies in the usage of a suitable classifier and the ability to design a flow feature set which is based on different types of traffic that can meet the classification specifications as shown in Figure 1.

#### 3.2.2. Spatial and Sequence Features

This part mainly analyzes the spatial and sequence features of network flow. In [16], the author proposed that the application of deep learning methods can realize the automatic extraction of network flow features, which is more suitable for the classification requirements than the manually and expert-originated features. In the article, authors used NN and SAE network to extract and classify the features of the processed network flow. Compared with the previous machine learning algorithms, the accuracy has been greatly improved. In the data preprocessing stage, the data packet obtained by the data link layer is used as the processing object, and packet is represented in the form of byte stream with the usage of the data packet extraction tool. After preprocessing, the data is sent to the neural network for training with the purpose of automatic extraction of feature.

**Figure 2:**
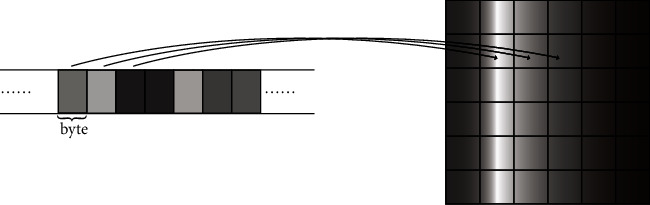
Schematic diagram of network flow transformed into a grayscale graph.

Inspired by the successful application of CNN in the field of image processing, the authors in [28] stated that the network data can be represented by a matrix as shown in Figure 2, that is, the flow sequence *F*_*p*_(*x*), transformed into a matrix *M*^*p*^, can be expressed as

(1) $$\begin{array}{c} F_{p}\left(x\right)⟶M_{ij}^{p}\left(n\times n\right),x=n\times i+j\text{.} \end{array}$$

In this way, there is a one-to-one correspondence between the specific matrix *M*^*p*^ and the network flow *F*^*p*^(*x*). For each node *M*_*ij*_^*p*^ of the matrix, the value range is (0–255), which is the same as the range of each pixel value of the grayscale image, so there is a one-to-one correspondence between the matrix *M*^*p*^ and the gray image *T*_*p*_. The network flow classification is transformed into grayscale image classification and network flow feature extraction is transformed into grayscale image spatial feature extraction.

It should be noted that although the abovementioned network flow classification task can be transformed into image classification, the extracted features are only the feature representation in the network flow graph, not the characteristics of the network byte stream, however, graph structure information is still very useful for feature analysis in this paper. On the one hand, if the feature is a unique feature of the network flow, it must be expressed in the form of a specific pixel in the map to form a specific spatial structure and this is the basis for the CNN to extract the spatial features of the network flow. On the other hand, the area formed by the feature also reflects the focus of the model in the classification task, which provides a reference for the analysis of classification features in this paper.

*(1) CNN Model Construction*. CNN is widely used in the field of image pattern recognition. It is a kind of deep learning model which contains a large number of convolution operations. A complete CNN includes several convolutional layers (CONV), pooling layers (POOL), and fully connected layers (*FC*). The common architecture patterns are shown as follows.

(2) $$\begin{array}{c} INPUT⟶\left[\left[CONV\right]\times N⟶POOL\right]\times M⟶\left[FC\right]\times K\text{.} \end{array}$$

The parameters of the convolutional layer are composed of some learnable filter sets (convolution kernels), which can capture the image features of the previous output layer, and another layer, pooling layer which is mainly responsible for subsampling. At last, a set of fully connected layers are often used to capture high-level features of an input.

This article uses the above architecture to learn features and classify the processed network flow which contains four convolutional layers (conv2d), two pooling layers (max pooling), three dense connection layers (dense) and one flattening layer (flatten), each data transformation in the model uses a normalization process (batch normalization).

*(2) HAN Model Construction*. Compared with converting the network flow to the graph and extracting the spatial feature information to complete the classification task, it is more straightforward to use the recurrent network to classify the network flow and extract the sequence information features of the network flow. The experiments in this section refer to the processing ideas of [31], mainly classify the network flow through the hierarchical attention network (HAN) [32], and display the feature distribution characteristics in the classification process through visualization technology.

**Figure 3:**
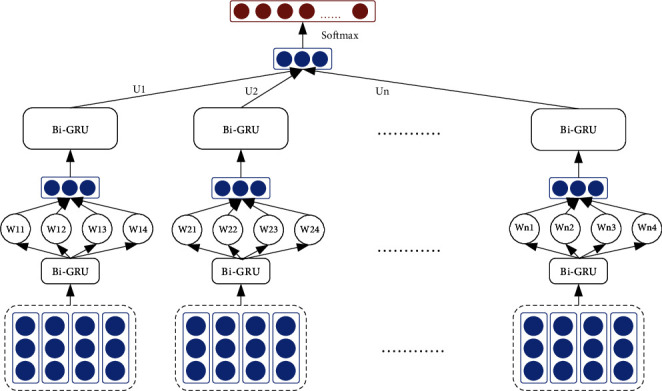
HAN for network flow classification.

This section adopts the byte-packet-stream processing mode, that is, a network flow label corresponds to a three-level data flow, this obvious hierarchical data structure is similar to the structure of token-sentence-article. The processing mode of network flow classification is shown in Figure 3.

This experiment uses the USTC dataset and divides the dataset into training set, validation set, and test set, which account for 60%, 20%, and 20% of the resampling data set, respectively.

Table 3 lists the classification results of the two models on the USTC test set. It can be seen from table that the two types of models have achieved high accuracy on the classification task, and the models perform well.

**Figure 4:**
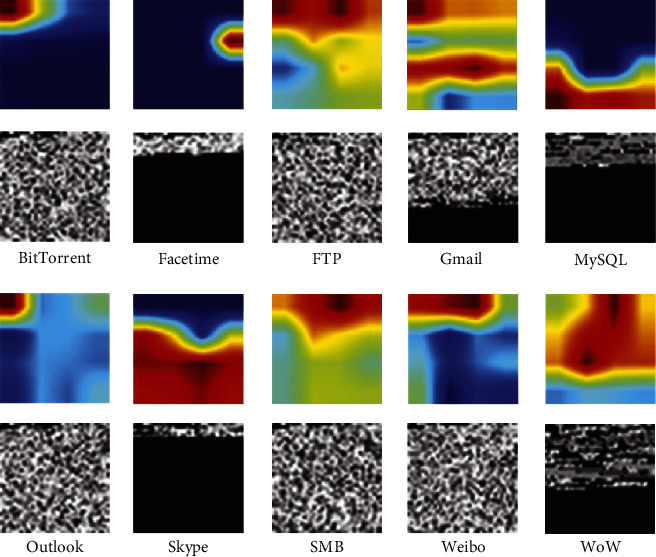
Class activation map for network flow classification.

This study randomly selects 120 samples in the test data for testing and uses Grad-CAM [33] technology to visualize spatial features. The visualization results are shown in Figure 4; among them, red represents the feature with higher activation degree and blue with lower activation degree, and in the grayscale image, 0 represents black and 255 represents white. Through visualization, we can see the distribution of important features of network flow classification in the CNN model.

From the class activation map, it can be seen that most of the features involved in network map classification are concentrated in the network flow header information, some of the features used for classification are concentrated in the tail of the data, and a small part of the map information also includes features in the middle. From the comparison of the original grayscale images, it can be seen that for MySQL, the black tail indicates that there is no network flow data in the current area, and the reason why the data information is not used for feature extraction is that MySQL does not have a unified representation in the grayscale image space., that is, feature extraction is more difficult. Compared with the information in the data, the structural information displayed by the map itself is more prominent and obvious, so the features used for classification are concentrated in the tail. A small number of features in the middle also show this feature distribution. For example, the middle features of Gmail are mostly concentrated on the boundary between the blank data area and the data area, which also reflects the unique feature structure information of the graph. Furthermore, in the WoW class, although the network graph also presents obvious structural features, the structural features of the information features of the WoW class are more obvious than those of the blank data area.

Next experiment randomly selects 10 samples of each type of network flow for attention visual analysis of HAN model.  Table 4 lists the visualization results of 10 types of network flow in the data set. The packet attention column represents the weight value of the packets in the network flow (the shade of red indicates the value), and the byte attention column represents the internal data of each network stream in hexadecimal, and identifies the weight value corresponding to each byte, in which blue indicates that the weight is larger, and green indicates less weight.

It can be seen from the table that when using sequence features to classify network flows, it shows different characteristics from spatial features in convolutional networks. When there are multiple data packets in the same flow, there is a specific packet that has a greater impact on the final classification, but the sequence characteristics of other packets with less impact are similar to those of packets with greater impact. As shown in the table, the information column lists the bytes with larger weights. The top ones include tcp.option_kind, tcp.option_len, ip.hdr_len, ip.len and other bytes. These features are related to network flow environment variables.

By visualizing the features of the two types of models, it can be found that the spatial features based on the network flow graph are similar to the network flow sequence features. From the perspective of feature distribution, the features with high weights are located in the first half of the network flow data. At the same time, there are differences between the two types of features. When the spatial features of the data are not enough to distinguish the types of network flows, the model prefers to use the spatial structure information of the graph, but the sequence information can only be extracted from the network flow itself. Therefore, both spatial features and sequence features can be used for network flow classification tasks, and the two types of features are distinguished from each other.

### 3.3. Proposed Classification Method

After obtaining the spatial and sequential features of the network flow, a natural idea is whether the classification accuracy can be improved if both types of features are used in the classification task. Based on the above two types of models, this study proposes a multimodal framework that uses the two types of features to classify the various types of network flow.

In [22, 34, 35], the authors proposed that for the same network, finding the best set of auxiliary tasks will improve the traffic classification which should be treated similar to hyper-parameter tuning. The abovementioned idea of multitask training can be expressed as: for the same network, using similar tasks to train separately, which can improve the efficiency of the network to complete the final task. Then, on the contrary, for the same task, there is a way to use different combination of methods to improve training efficiency of the target task.

#### 3.3.1. Multimodal Network Flow Classification Model

Through the abovementioned analysis, we can extract spatial features through the CNN model and sequential features through the GRU model to build the multimodal framework. There are two main ways to build the framework.

**Figure 5:**
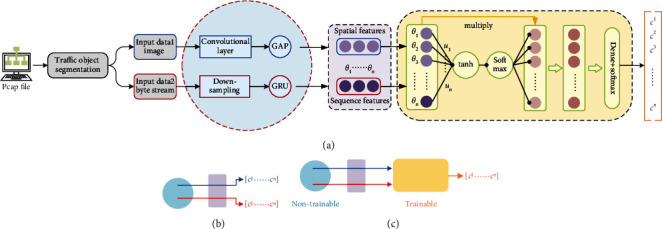
The framework based on pretraining. (a) Pretraining model, (b) pretraining, and (c) parameter training.

*(1) Model Pretraining (Model A): The Framework Based on Pretraining*. The framework based on pretraining refers to: train the networks, respectively, and select the characteristics preliminary, then integrate the selected features of each network and send them to the secondary network for further screening, as shown in Figure 5.

The model in Figure 5(a) shows the structure of the multimodal classification model based on pretraining. First, the pcap file is segmented, and the data is preprocessed to form the input data format file (image and byte stream), and then sent to the the convolutional layer and the downsampling layer to extract and simplify the data features. The spatial features and sequence features of the network flow are extracted through the GAP layer and the GRU layer, respectively, and the extracted two types of features are then sent to the feature fusion module to form fusion features. The dense layer and softmax are used for output of classification.

Figure 5(b) shows that the features used in Figure 5(a) are extracted from the two submodels through pretraining.  Figure 5(c) shows that the model parameters in the first half of the model in Figure 5(a) are actually frozen and do not participate in the training of the entire model. The training part is mainly the parameters of the feature fusion part.

Strategy of combination: during the training processing, the features of the same type will be completely extracted layer by layer, at last, it will focus on the features useful for the task. For example, in the field of image recognition and classification, with the usage of heat map [33], it is easy to find the important feature which will be helpful for the final task. However, this type of feature set is still redundant for the entire training task. Each feature in the mixed feature set does not necessarily contribute to the final classification task and same feature may have different weights in different models. Therefore, it is necessary to further filter the extracted features. In order to filter the combined features, this article adds a layer of weight learning to the second step to realize the automatic assignment of the weight of each feature.

Assuming that the feature set is *θ*, and each feature is represented as *θ*_*i*_, the weight of *θ*_*i*_ can be calculated by the following equations:

(3) $$\begin{array}{c} w_{i}=\tanh\;\left(u_{i}\cdot \theta +b_{w}\right)\text{,} \end{array}$$

(4) $$\begin{array}{c} \alpha_{i}=\frac{\exp\left(w_{i}\right)}{\sum_{t}\exp\left(w_{i}\right)}\text{.} \end{array}$$

We calculate the dot product of the weight and the original feature and recombine it into a new feature set *θ*^*τ*^:

(5) $$\begin{array}{c} \theta^{\tau }=concatenate\left[\alpha_{i}\cdot \theta_{i}\right]\text{.} \end{array}$$

The classification task is implemented through a fully connected layer and softmax layer.

Based on the abovementioned the framework design, on the one hand, it is beneficial for the framework to filter feature sets automatically, which meets the processing requirements to allocate different feature sets for different types of network flow. On the other hand, by analyzing the attention of each feature, we can further study the importance of each feature to the classification task.

In summary, two types of features are extracted from the trained network previously, after integrating the extracted features, they are sent to the second learner for training again to complete the final task. This training method needs to be divided into two steps. The performance of the first step learner directly affects the second.

**Figure 6:**
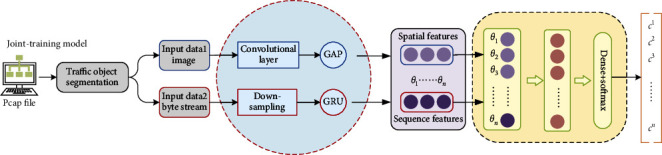
The framework based on joint-training.

*(2) Model Joint-Training (Model B): The Framework Based on Joint-Training*. The main idea of this method lies in the combination of models, that is, the characteristics learned by the two types of models are directly combined in one network to construct a wide and deep large-scale network model as shown in Figure 6. The extracted features are more diverse and accurate than that of a single network, and can be directly used for classification tasks. The framework only needs a single-step training to obtain a useable model.

*(3) Comparison of Two Types of Frameworks*. Although the abovementioned models are based on the idea of integration of mixed feature, they are very different in the way of framework construction and training method.

*Framework Construction.* Model pretraining can actually be divided into three models, including two basic models, namely, the CNN model for extracting spatial features and the GRU model for extracting sequential features, and a secondary model. In the secondary model, it is necessary to design a proper extraction strategy of input data. In this study, the attention-like mechanism is used to realize the automatic learning of feature weights. Model joint-training is one model essentially. The characteristic of model joint-training is wide and deep, that is, in terms of width, the integration of multiple models is adopted to expand the longitudinal direction of the framework, and for the depth, the feature extracted by the basic model is relearned and trained to expand the horizontal direction of it.

*Training Method*: Model pretraining is divided into two steps in the training method. The first step is to train basic models to complete the preliminary feature extraction. The second step is to send the extracted features to the secondary model to complete the final training task. In fact, the above training can be referred to as one training step, as the training task is completed on the secondary model finally, not on the basic model. Model joint-training only needs one step for training, that is, sending the data to the network for training directly, which is an end-to-end training model actually. Considering training task in practice, although model pretraining needs to complete the training of three models, the basic model can be trained in parallel and separately, which is more flexible for actual operations. In contrast, it seems that model joint-training only needs one step to train, actually, the time and hardware parameters required in the training environment are higher than those of model pretraining due to the high complexity of joint training and the inability to perform parallel processing.

Notably, it is worth to mention that the multimodal framework idea is different from the ensemble strategies, which is widely adopted in machine learning competitions. Ensemble strategies improve the efficiency of the ensemble model through reducing the deviation and variance between basic models by adjusting the data set or combination of training result, like boosting integration [36, 37], bagging integration [38] and stacking integration. The multimodal framework is the integration of extracted patterns from different dimensions to produce the final result. It is to integrate the data from different perspectives to make the collected information more comprehensive with assigning different weights according to different features automatically that making the framework more robust and efficient.

#### 3.3.2. The Framework Cross-Validation Criteria

In order to verify the reliability of the mentioned framework, we used *k*-fold cross validation on the training data to conduct experiments on different data sets. Specific algorithm 1 implementation is as follows.

#### 3.3.3. The Framework Evaluation Criteria

To evaluate the performance of the multimodal framework, we have used accuracy (*Ac*), recall (*Rc*), precision ( Pr ), and *F*_1_ score (*F*_1_) metrics [39–42]. The abovementioned metrics are described mathematically as follows:

(6) $$\begin{array}{c} Ac=\frac{TP+TN}{TP+TN+FP+FN}100\%\text{,} \end{array}$$

(7) $$\begin{array}{c} Rc=\frac{TP}{TP+FN}100\%\text{,} \end{array}$$

(8) $$\begin{array}{c} \mathrm{Pr}\;=\frac{TP}{TP+FP}100\%\text{,} \end{array}$$

(9) $$\begin{array}{c} F_{1}=\frac{2\cdot Rc\cdot \mathrm{Pr}}{Rc+\mathrm{Pr}}100\%\text{,} \end{array}$$

where TP, TN, FP, and FN stand for true positive, true negative, false positive, and false negative, respectively.

## 4. Experiment

The experiment is divided into four steps.

- Converting raw data to trainable data
- Network flow spatial feature learning, mainly using CNN to classify the grayscale images
- Network flow sequential feature learning, mainly using GRU network to classify network flow digital sequences
- Hybrid feature learning, which uses the multimodal framework to classify network flow

The TensorFlow [43] is used as an experiment software framework that runs on Windows 10 home edition with Intel(R) Core (TM) i5-9300H CPU @ 2.40 GHz and 8 GB memory. An Nvidia GeForce GTX1650 GPU is used as an accelerator. The mini-batch size is 256 and the cost function is categorical cross-entropy. Adam optimizer built-in TensorFlow is used as an optimizer, training time is about 70 epochs.

### 4.1. Dataset and Preprocessing

In this article, we used the ISCXVPN2016 dataset [29] mentioned in A. In order to better compare the experimental results, this study relabels the dataset according to the classification method of the literature [44]. Under-sampling is also applied according to the number of data set. Sampling is a simple method to tackle this problem. Hence, to train the proposed framework, using the under-sampling method, we randomly select samples of major classes until the classes are relatively balanced.

The dataset above is obtained from the data link layer. From hierarchical perspective, at the data link layer, the frame header information contains physical connection information, such as MAC address and other protocol content. The network transmission layer also contains IP address information. These data play a key role in network stream transmission, but they cannot provide any valuable information in the field of network flow classification and even training networks will use the address information to classify the network flow, which is ridiculous in practice. Therefore, in the data preprocessing part, the MAC and IP addresses are directly removed to eliminate the impact on the training task due to different addresses.

The second step is file cleaning, which is to clean up duplicate network stream files and empty files to avoid bias when training the network.

Finally, due to the need of using deep learning network to train the data, the data length standard needs to be unified. TCP and UDP protocol headers have of different length. In order to unify the length of the transport layer, we inject zeros to the end of UDP segment's headers to make them equal with TCP headers. Finally, according to the literature [16], most of the valuable information is at the header of payload data. In this article, the first 1225 bytes (35 × 35) are intercepted as the research object in the ISCXVPN2016 dataset to balance the accuracy and simplicity of the experiment.

Through the analysis of the number of data samples, we found that it is very different with the number of samples of various types of data. In the literature [45, 46], experiment results show that the performance of the learner will decrease due to the unbalanced number of samples, and this study will adopt a random sampling method until the number of various flows is relatively balanced. According to the network flow generated by different application types, this study relabels the data set and divides the network flow into 17 categories, as shown in Table 5.

The dataset is divided into the training set, validation set, and test set, which respectively account for 60 %, 20 % , and 20 % of the resampled dataset.

### 4.2. Results of the Multimodal Framework

**Figure 7:**
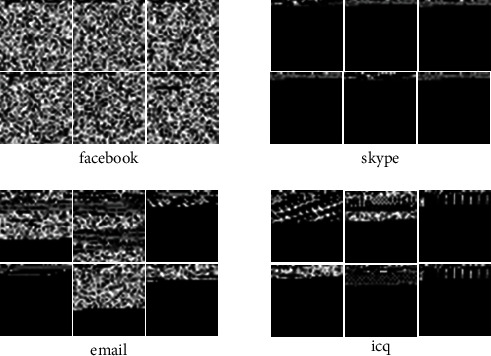
Grayscale images of different types of network flows.

Since the framework combines CNN and GRU, it is necessary to convert the network flow into trainable grayscale images and digital sequences, respectively. In the spatial and sequential features section above, we discussed how to convert the network flow into a matrix and then into a grayscale image in [28], which is the input into CNN for training.  Figure 7 shows part of the grayscale images of ISCXVPN2016 data set after network flow conversion.  Figure 7 lists visual pictures of part of the network flows which shows vividly that for different types of network flow, it is distinguishable for texture characteristics of the picture. The above analysis can infer that after the network flow is converted to grayscale image, the different network flows have distinguishable features, and the same types have consistent ones.

In the part of input data of GRU, similar to the text processing method, the value corresponding to each byte of the network flow is similar to the token after the text tokenization which can be called network flow token, then associating it with vector by using embedding method. These vectors are combined into a sequence tensor, which is input into the GRU.

Table 6 shows the performance of four types of models (CNN, GRU, model pretraining, model joint-training) on the test set. Experiments show that the multimodal framework has entirely extracted network flow characteristics to distinguish each application accurately.

**Figure 8:**
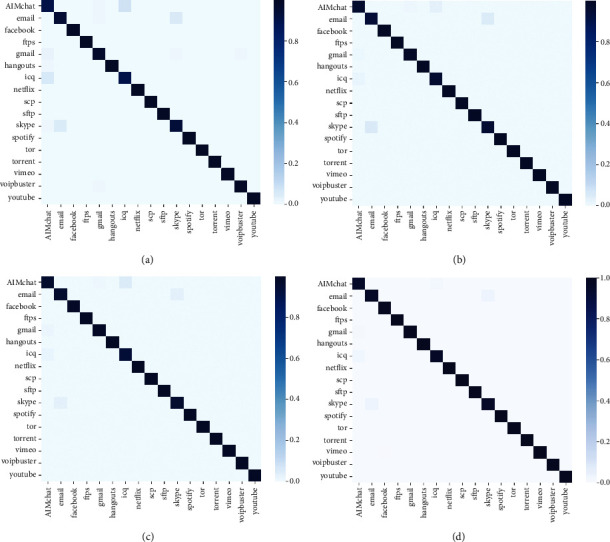
Heat map of test results for each model; the abscissa represents the true label of each type of network flow, and the ordinate represents the predicted label. The color represents the predicted probability. (a) CNN model, (b) GRU model, (c) model pre-training, (d) model joint-training.

**Figure 9:**
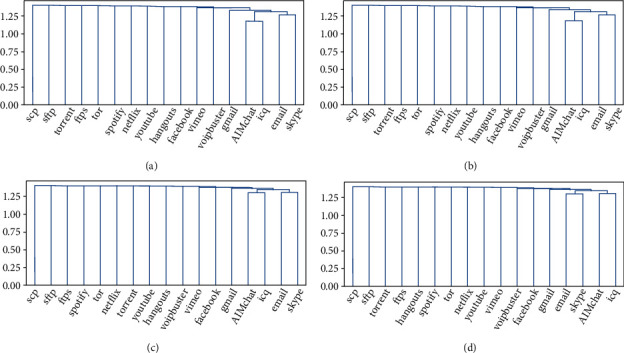
Hierarchical cluster diagram; the tree structure reveals the distribution of network flow clustering. (a) CNN model, (b) GRU model, (c) model pre-training, (d) model joint-training.

In order to show the experimental results of the model on the test set in more detail, this study draws a heat map based on the prediction results of each type of network flow. At the same time, hierarchical clustering is used to analyze the relationship of each type of network flow [47]. This method uses Euclidean distance as the distance metric, average as the agglomeration method, and reveals the different relationships among the different types of network flows. Here, we just show the result of the model pretraining and joint-training, as shown in Figures 8, 9. The figures indicate four models have achieved high classification accuracy. In particular, model pretraining and model joint-training have similar results in accuracy and are better than the basic model. The clustering reveals that there are similarities between AIMchat and ICQ, skype and e-mail. Furthermore, there are similarities between Gmail, AIMchat, and ICQ. This is consistent in practice, as both AIM and ICQ provide online chat functions, and skype and e-mail also provide chat services based on the online. The conclusion is similar to [44] but more accurate which reveals the true relationships among the different types of network flows. Network flow classification shows a certain relationship because of the functional similarity of the applications abovementioned, which just shows that the network flows are classified on the basis of extracting features accurately.

### 4.3. Comparison

In this section, we compare the experimental results of network flow classification based on the ISCXVPN2016 dataset in recent years. Among them, Yamansavascilar et al. [48] used the *k*-NN method to classification, Lotfollahi et al. [44] used a method called “Deep Packet,” namely SAE and CNN to classify network flow. It can be found from Table 7 that both model pretraining and model joint-training outperform two methods above.

It should be noted that Yamansavascilar et al. used machine learning methods to implement classification based on manually and expert-originated feature sets. Lotfollahi M et al., model pretraining and model joint-training use deep learning methods. This article has analyzed the shortcomings of classification based on manually and expert-originated features, and automatic extraction of network flow features based on deep learning is more suitable for practical applications with the development of intelligence.

According to [44], we compared the results of the ISCXVPN2016 data set based on different machine learning methods, where the decision tree depth parameter is 2, random forests is four, regression (with *c* = 0.1), and naive Bayes with default parameters. As shown in Table 8, combined with Table 6, we can find that the classification based on deep learning is better than that of various machine learning. This shows the power of deep learning tools, especially in processing big data tasks and is consistent with the analysis results of the III-B1.

**Figure 10:**
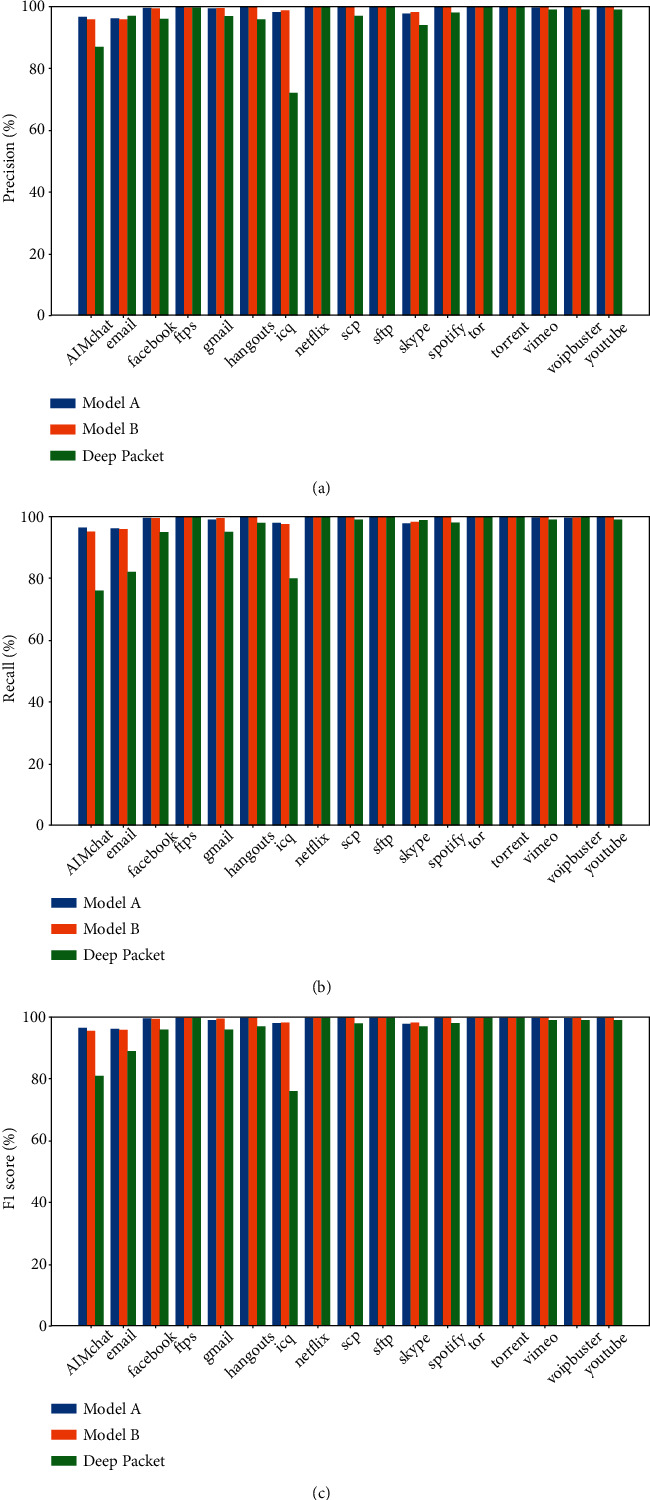
Comparison of each class experiment result with the benchmark model. (a) Precision of models, (b) recall of models, and (c) F1 score of models.

Figure 10 further shows the experimental results based on the deep learning on the test set. It indicates that the two types of the multimodal framework outperform the method based on “Deep Packet” in network flow classification.

### 4.4. Discussion

In this part, we try to explore why the multimodal framework has better performance. Basic learners with relatively good performance observe the data from different dimensions and obtain part of the information of the truth, but not all of the information. Only by gathering all kinds of information together can we get a more accurate description of the data. In order to ensure the effectiveness of the multimodal framework, the key lies in the diversity of the learners. The deviation of different learners is from different perspectives; after integrating the different learners, the framework will cancel each other in these deviations, so the result is more stable and accurate.

The abovementioned two models are the CNN and GRU model for extracting spatial and sequential features. The two types of models are different, so the deviations in the classification can be offset by each other, so as to achieve more stable and accurate classification results.

**Figure 11:**
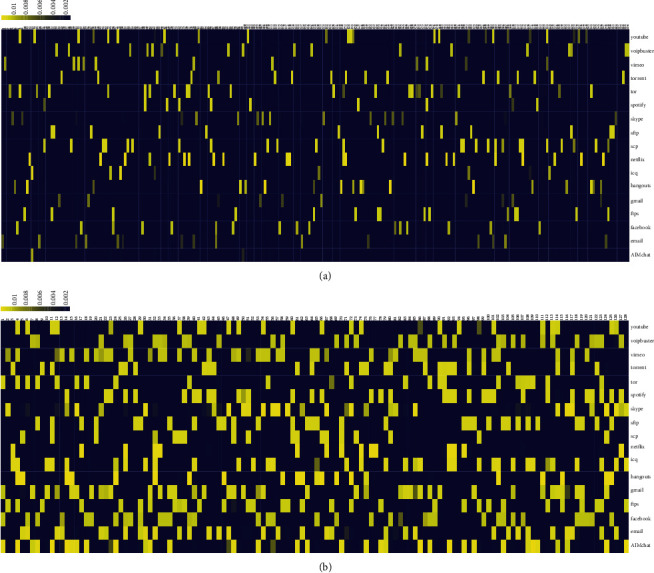
Distribution map of attention value of each type of network flow; the ordinate is the network flow type, and the abscissa is the number of extracted feature. (a) The spatial feature extracted by CNN; (b) the sequential feature extracted by GRU; the intensity of the color indicates the contribution of a certain feature to the final classification result.

Figure 11 is our attention analysis based on the features extracted by model pretraining. The two types of basic models are integrated into input data after extracting 256 and 128 features of each training object, and then sent to the secondary learner for training. As can be seen from Figure 11, on the one hand, the proportion of sequence features used by secondary classifiers is much larger than that of spatial features, because the classification accuracy of GRU network using sequence features is higher than that of CNN network classification. On the other hand, from the perspective of usage of the overall feature, in the secondary classifier, both spatial and sequence features participate in the final classification task, which indicates that the above two types of features both contribute to the classification task and promote the classification efficiency of the model.

**Figure 12:**
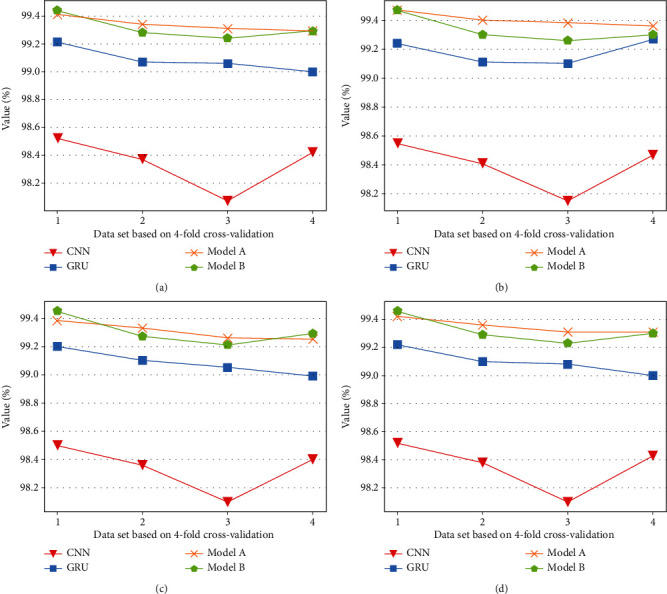
Experiment results based on the method 4-fold cross validation. (a) Accuracy of models, (b) Precision of models, (c) recall of models, (d) F1 score of model.

Figure 12 shows the result of models trained on the different data based on *k*-fold cross-validation. It can be seen from the figure that the *Acc* scores of the result of the CNN model vary from 98.07 % to 98.52 %, and the scores of the GRU model are (99.01 %-99.21 %). The scores of model pretraining are (99.29 %-99.41 %), and the values of model joint-training are (99.23 %-99.45 %). Although the performance of the basic model is slightly different on different data sets, the multimodal framework has improved the performance of the basic model.

**Figure 13:**
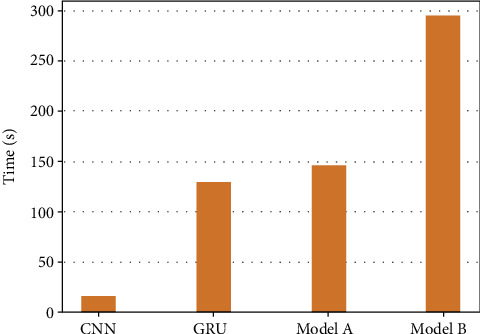
Time consumption of different models.

In III-C1, we compared the two types of models and found that compared with model pretraining, model joint-training requires higher training conditions and more time.  Table 9 shows the specific parameters of each model.  Figure 13 shows how much time it needs to build a model on the training set of 113004 flows and validation set of 37668 flows in each epoch based on the same training environment mentioned above. As shown in the figure, model pretraining requires less training time than model joint-training, and the basic models in model pretraining can be processed in parallel. It seems that the model pretraining has more advantages in practice. But the latter one is an end-to-end model, if the size of sample data is relatively insufficient and data enhancement is allowed for training tasks, model joint-training is more powerful, and model pretraining is limited to the dataset used by the basic model. From this perspective, it is more flexible in selection of data set comparing model joint-training with model pretraining. Therefore, in practice, it is necessary to select the proper model according to the specific training task.

## 5. Conclusions

To solve network security problems, this study proposes a new method of network flow classification based on deep learning: the multimodal framework which is based on pretraining and joint-training, respectively. To the best of our knowledge, this is the first time that such a framework has been proposed in the field of network flow classification. Experimental results show that the framework outperform similar work done in recent years based on the data set ISCXVPN2016. At the same time, the multimodal framework is a deep learning network, which is handy in processing big data. More data is conducive to the improvement of the framework performance, moreover it can realize the automatic extraction of network flow features, saving a lot of manpower and time which has good practical significance. We believe that the multimodal framework is a meaningful attempt in the field of network security, and it is also very useful for the construction of a human physical and mental health system.

In the next step, we can adopt more methods suitable for network flow classification and expand the framework to build a better classification model. At the same time, it is possible to explore new network for network flow classification tasks. For example, in [23], the author proposed that the capsule network can improve the efficiency of classification tasks, which can be considered as a new direction for future research. With the continuous improvement and use of transformer in the field of feature extraction, it also has great inspiration for the feature extraction of network flow.

In the experiment, we extracted the high-level information features of the network flow and studied the impact of feature extraction on the classification task. In order to fit the experimental results, high-level information may lose some important information, causing the extracted features to not fully reflect the unique information of the network flow. At the same time, due to the usage of CNN, it is difficult to extract the global information of the features, and it may also affect the classification results. New technologies, such as the Conformer structure [49], can be applied to the field of network flow classification; moreover, we can explore other feasible technologies.

Finally, this article mainly discusses the feasibility of the framework using unencrypted datasets to conduct experiments. In practice, as information security has received much attention, network encryption has become the mainstream. The next step is to conduct experiments based on the encrypted network flow for better application in practice.

## Figures and Tables

**Algorithm 1:**
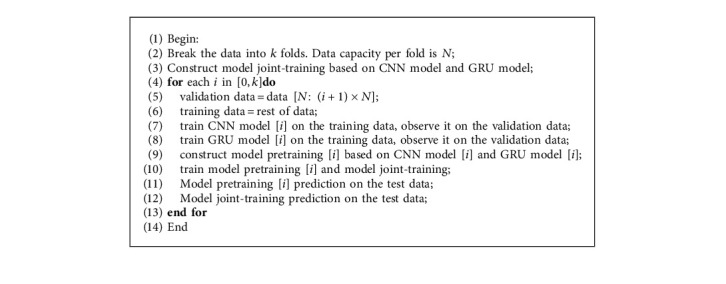
*k*-fold cross validation used to test the stability.

**Table 1:** List of the acronyms used in this study.

Acronym	Definition
QoS	Quality of service
RNN	Recurrent neural network
DPI	Deep packets inspection
GRU	Gated recurrent unit
FCBF	Fast correlation-based filter
CV	Cross validation
LDA	Linear discriminate analysis
CONV	Convolutional layers
SAE	Sparse autoencoder
NN	Neural network
FC	Fully connected layers
CNN	Convolutional neural network
ML	Machine learning
LSTM	Long and short-term memory
SFS	Sequential forward selection
RTT	Round-trip time

**Table 2:** Summary of methods in the literature employing machine and deep learning models.

Ref.	Model	Data	Eva metrics	Contribution
[12]	Naive bayes	—	Accuracy	A vast improvement over traditional techniques

[13]	Nearest neighbour (NN) and linear discriminant analysis(LDA)	WAND	Error rate	Using traffic traces from a variety of network locations, demonstrate the feasibility, and potential of the approach

[14]	Noise elimination, random forest(RF)	ToN and ISP	Accuracy, *F*1	The framework delivers consistently superior performance to other traffic classification schemes in the presence of unclean training data

[15]	A classifier based on Weka's classifiers library	—	Recall, precision, accuracy	Authors suggest a fingerprint that is based on zero-length packets, hence enabling a highly efficient sampling strategy

[16]	NN and SAE	TCP flow data	Precision, recall	The approach solves the problem of nonautomation and poor adaptation in traditional ways

[17]	CNN	Data including 5 protocol and 5 application	Accuracy	Propose a nearly end-to-end framework for online IP traffic classification

[19]	LSTM	Real server-generated traffic	Accuracy	The LSTM NNs prove to be a highly efficient computational model capable of solving real server-generated traffic

[20]	CNN and RNN	Internet of things traffic	Recall, precision, accuracy, *F*1	The study shows the performance of CNN and RNN models and a combination of them

[21]	CNN and LSTM	Mobile traffic	Recall, precision, accuracy, *F*1	Introduce two deep learning models for mobile app identification

[22]	RF, CNN, RNN, multitask learning	QUIC and ISCX	Accuracy	Multitask learning approach out-performs, or performs as accurately as the transfer learning

[23]	Capsule network	UTSC-2016	Recall, precision, accuracy, *F*1	This study proposed an end-to-end traffic classification method and used the capsule network model for traffic classification

[24]	CNN, LSTM, SAE	Encrypted mobile traffic	G-mean, accuracy, *F*1	This study provided a wide experiment analysis based on multimodel framework (CNN + LSTM) for classification of encrypted mobile traffic

[26]	RNN, autoencoder	Encrypted traffic	True positive rate, false positive rate, FTF	This study provided the framework containing a multilayer structure which can explore sequential characteristics deeply and import the reconstruction mechanism which can enhance the effectiveness of features

[25]	CNN, RNN	ISCX VPN-nonVPN	Accuracy, *F*1	This study proposed a novel multimodal multitask deep learning approach and DISTILLER classifier, it can solve different traffic classification simultaneously

[27]	CNN, RNN	MIRAGE-2019	Accuracy, *F*1, G-mean, precision	This study used explainable artificial intelligence to improve multimodel behavior, the experiment results showed that the proposed method provide global interpretation, rather than sample-based ones

**Table 3:** Accuracy (%) of models on the test set.

Type	HAN	CNN
BitTorrent	99.92	99.75
FaceTime	99.92	100
FTP	100	99.92
Gmail	99.92	99.33
MySQL	100	100
Outlook	100	99.92
Skype	100	100
SMB	100	100
Weibo	100	99.92
WoW	100	100
Average	99.98	99.88

**Table 4:** HAN attention visualization.

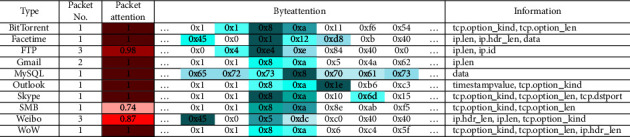

**Table 5:** Type of network flow in ISCXVPN2016.

AIMchat	E-mail	Facebook	ftps	Gmail
Hangouts	icq	Netflix	Scp	Sftp
Skype	Spotify	tor	Torrent	Vimeo
Voipbuster	Youtube			

**Table 6:** Four models' performance for the network flow classification.

Model	*Ac*(%)	Pr(%)	*Rc*(%)	*F*_1_(%)
CNN	98.52	98.55	98.50	98.52
GRU	99.21	99.24	99.19	99.22
Model *A*	99.41	99.46	99.38	99.42
Model *B*	99.45	99.47	99.45	99.46

**Table 7:** The comparison between the multimodal framework and other methods on the “ISCXVPN2016” dataset.

Study	Metric	Result (%)	Alg.
Yamansavascilar et al.[48]	Accuracy	94	*k*-NN
Deep Packet (2020)		98%	CN
The multimodal framework		99.41	Model pretraining
The multimodal framework		99.45%	Model joint-training

**Table 8:** The comparison between the multimodal framework and other machine learning methods on the “ISCXVPN2016” dataset.

Method	Pc	Pr	F1
Decision tree	0.90	0.90	0.90
Random forests	0.91	0.90	0.90
Logistic regression	0.91	0.91	0.91
Naive Bayes	0.40	0.34	0.26

**Table 9:** Comparison of model parameters.

Model	CNN	GRU	Model *A*	Model *B*
Params	424,145	150,673	346,769	589,713

## Data Availability

The datasets used in this study are available on public repositories.
